# Supplementary material for: Oxytocin Reduces Subjective Fear in Naturalistic Social Contexts via Enhancing Top‐Down Middle Cingulate Amygdala Regulation and Brain‐Wide Fear Representations
Source: Adv Sci (Weinh). 2025 Jul 18;12(38):e03251. doi: 10.1002/advs.202503251 (PMC12520527; doi:10.1002/advs.202503251)
Supplement: Supplementary file 1 — Supporting Information [file ADVS-12-e03251-s001.docx]

Supplementary Information

Oxytocin reduces subjective fear in naturalistic social contexts via enhancing top-down middle cingulate amygdala regulation and brain-wide fear representations

Authors

Kun Fu^1,2^, Shuyue Xu^3,4^, Zheng Zhang^1,2^, Dan Liu^1,2^, Ting Xu^1,2^, Yuan Zhang^1,2^, Feng Zhou^5,6^, Xiaodong Zhang^1,2^, Chunmei Lan^1,2^,Junjie Wang^1,2^, Lan Wang^1,2^, Jingxian He^1,2^, Keith M Kendrick^1,2^, Bharat Biswal^2,7^, Zhen Liang^3,4^*, Weihua Zhao^1,2^*, Benjamin Becker^1,2,8,9,10^*

Affiliations

^1^ The Center of Psychosomatic Medicine, Sichuan Provincial Center for Mental Health, Sichuan Provincial People’s Hospital, University of Electronic Science and Technology of China, Chengdu, China

^2^ MOE Key Laboratory for Neuroinformation, School of Life Science and Technology, University of Electronic Science and Technology of China, Chengdu, China

^3^ School of Biomedical Engineering, Medical School, Shenzhen University, Shenzhen, China

^4^ Guangdong Provincial Key Laboratory of Biomedical Measurements and Ultrasound Imaging, Shenzhen, China

^5^ Faculty of Psychology, Southwest University, Chongqing, China

^6^ Key Laboratory of Cognition and Personality, Ministry of Education, Chongqing, China

^7^ Department of Biomedical Engineering, New Jersey Institute of Technology, Newark, USA

^8^ State Key Laboratory of Brain and Cognitive Sciences, The University of Hong Kong, Hong Kong, China

^9^ Department of Psychology, The University of Hong Kong, Hong Kong, China

^10^ Lead contact

*Corresponding authors

bbecker@hku.hk (B. Becker, lead contact)

janezliang@szu.edu.cn (Z. Liang)

zarazhao@uestc.edu.cn (W. Zhao)

Supplementary Methods

**Participants of pilot study**

In total an independent sample of 20 healthy young participants (10 females; age = 22.65 ± 2.01) with normal or corrected-normal vision from the University of Electronic Science and Technology of China were recruited in the study to evaluate the potential clips for the later fMRI study. All the participants did not regularly watch horror movies or were particularly timid.

**Exclusion criteria of participants of fMRI study**

Exclusion criteria of participants comprised a history of head injury, color blindness, current or regular substance or medication use, current or history of medical or psychiatric disorders, and any contraindications for oxytocin or MRI.


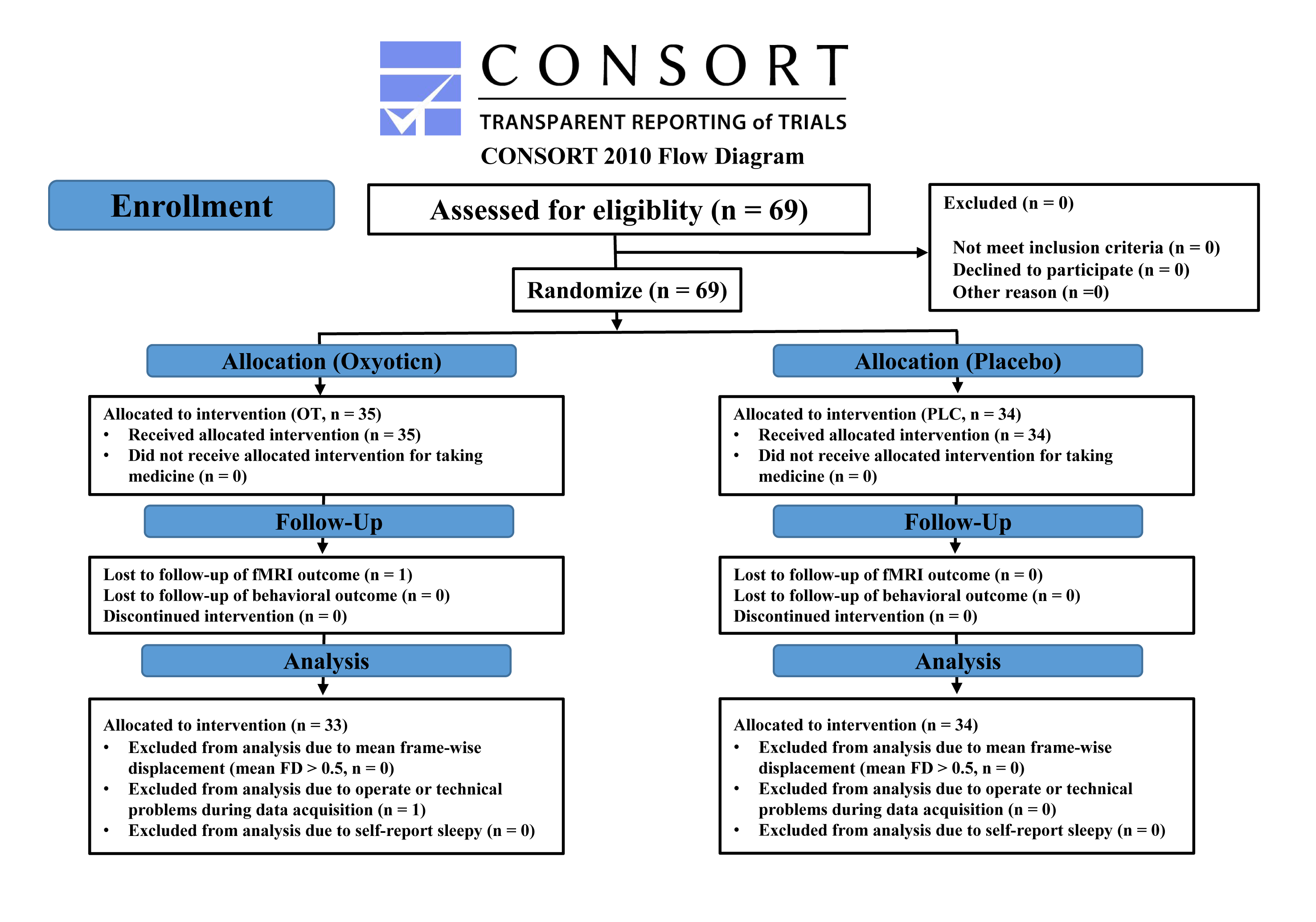


Fig. S1 CONSORT Flow Diagram.

MRI acquisition and preprocessing

Functional MRI data was acquired using a T2*-weighted echo-planar imaging (EPI) pulse sequence (repetition time = 2000 ms, echo time = 30 ms, 36 slices, slice thickness = 3.8 mm, no gap, field of view = 200 × 200 mm, resolution = 64 × 64, flip angle = 90°, 3.125 × 3.125 × 3.8 mm voxels, axial scan plane). To improve spatial normalization and exclude participants with apparent brain pathologies a high-resolution T1-weighted image was acquired using a 3D spoiled gradient recalled (SPGR) sequence (176 slices, repetition time = 8.22 ms, echo time = 3.15 ms, field of view = 256 × 256 mm, resolution = 256 × 256, flip angle = 8°, 1 × 1 × 1 mm voxels).

The MRI images preprocessing included 1) Spatial normalization to the ICBM 152 Nonlinear Asymmetrical template version 2009c was performed through nonlinear registration with the antsRegistration tool of ANTs v2.3.3 ^1^, using brain-extracted versions of both T1w volume and template. 2) Brain tissue segmentation of cerebrospinal fluid (CSF), white matter (WM) and gray matter (GM) was performed on the brain-extracted T1w using FAST (FSL v6.0.5.1) ^2^. 3) Before the automated preprocessing, 5 initial volumes of fMRI data were removed to allow for image intensity stabilization. 4) Next, functional data was slice time corrected using 3dTshift from AFNI ^3^ and motion corrected using mcflirt (FSL v6.0.5.1). This was followed by 5) co-registration to the corresponding T1w using boundary-based registration ^4^ with six degrees of freedom, using FLIRT (FSL). 6) Motion correcting transformations, 7) BOLD-to-T1w transformation and 8) T1w-to-template (MNI) warp (interpolated to 2 mm isotropic voxels) were concatenated and applied in a single step using ants ApplyTransforms (ANTs v2.3.3) using Lanczos interpolation.

Preprocessed images were spatially smoothed using an 8-mm full-width at half maximum (FWHM) Gaussian kernel in SPM 12.

Definiton of ROIs

The template utilized in this study is in line with a previous study^5^ which comprises 400 cortical regions from the Schaefer atlas ^6^, 34 subcortical regions from the Melbourne subcortex atlas ^7^, and the reinforcement learning atlas (extended amygdala and hypothalamus) ^8^ and periaqueductal gray (PAG), brainstem, and cerebellar regions (n = 29) ^9^, resulting in a total of 463 regions.

Video clips materials evaluation procedure

The participants were required to watch 40 short video clips attentively and rate their feelings using a series of 9-point Likert scales while watching the videos on five dimensions after watching each video clip (see **Fig. S2**). The five rating scales included arousal (where 1 represents very calm; 9 indicatesextremely arousing), valence (where 1 represents very positive and pleasurable; 9 represents very negative), fear (where 1 represents no fear at all; 9 represents extreme fear), social (where 1 represents exceptionally low levels of sociability, with no social context at all; 9 indicates exceptionally high levels of sociability, with high levels of social context and interactions), and consistency (where 1 extermely low emotional consistency of the video clip; 9 represents a high level of emotional consistency across the movie clip).

To screen the stimulus materials that matched our experimental design, we sorted all the videos on each of the five rating dimensions from highest to lowest and selected the video clips for the subsequent MRI experiments (8 clips for each of the 4 conditions FS\FNS\NS\NNS). To further verify that the selected video clips could effectively evoke the corresponding emotions (FS\FNS\NS\NNS), we performed a series of repeated measures ANOVAs on each of the five rating dimensions of the selected stimulus materials, respectively.


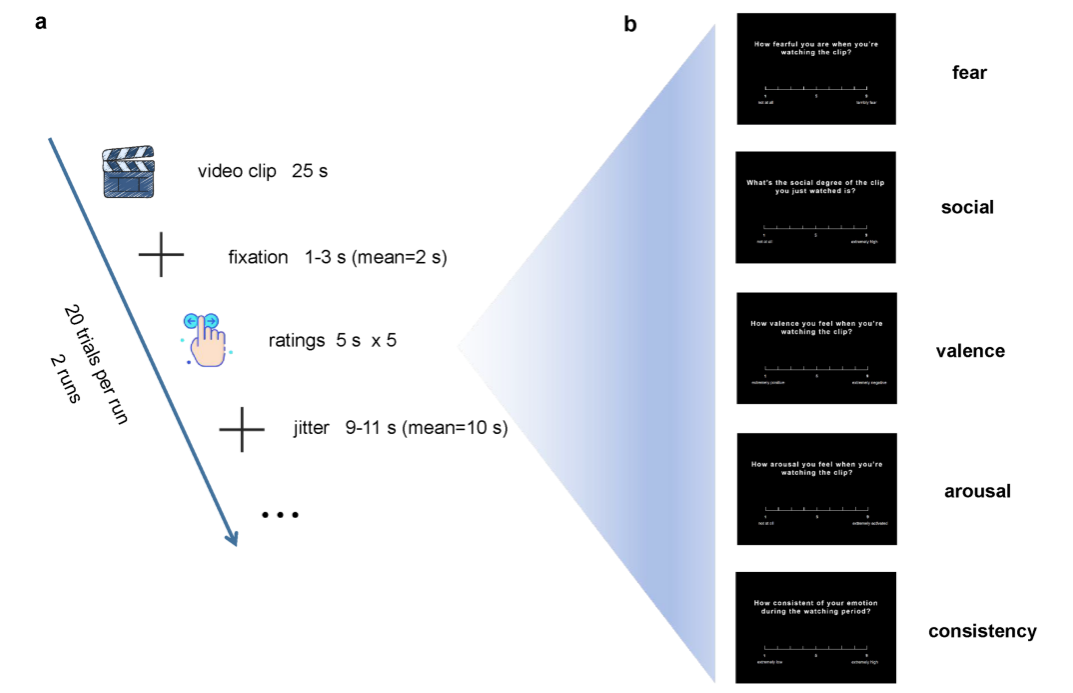


Fig. S2 Video clips materials evaluation procedure.

Key exclusion criteria for clip materials selection

Video clips for the fear condition were excluded if they (1) lacked fear-specificity, defined as more than one participant (5%) reporting the induction of non-fear emotions (e.g., disgust, sadness, happiness, joy) during the movie; （2）one ratings for arousal, valence or fear was below 4.5; (3) exhibited low emotional consistency (rating ≤ 6.5) over the duration of the clip (25s). Video clips for the neutral condition were excluded if they (1) induced fear\arousal ratings exceeding 4.5; (2) triggered positive or negative emotions during the watching period (valence ratings below 4 or exceeding 6, with 4-6 representing neutral); (3) exhibited low emotional consistency (rating ≤ 6.5) over the whole length of the clip. Following this exclusion process, 32 video clips were retained for the formal fMRI study.

The selected clips robustly induced subjective fear

To initially ensure that the selected video clips (8 clips in each condition) effectively induced the intended emotions (FS\NS\NS\NNS), a series of repeated measures ANOVA (sex was used as the independent variable given that the pilot study included male and female participants, while emotion and social were used as repeated variables) were conducted on five rating metrics in the data from the pilot study. Analyses were conducted using GraphPad Prism 9.0 (GraphPad Software, Inc., La Jolla, CA). These analyses were conducted on the data from the pilot study in an independent sample.

In addition, we further explored whether the selected clips engaged fear-related brain regions. To this end, we conducted univariate activation analyses based in the PLC-treated participants of the fMRI study. The corresponding univariate GLM model contained five regressors (FS\FNS\NS\NNS and the rating period, details see main manuscript) and the contrasts of interest modelled fear in social and non-social contexts separately (i.e., [FS], [FNS], [FS - NS] or [FNS - NNS]). Next, one sample t-tests were conducted on the second level analyses.

**Effects of OT on large-scale brain network functional connectivity modelled as interaction contrast**

We initially modeled the network level effects by subtracting the Z-values matrix for the two social conditions (ZFS-NS\ZFNS-NNS) and by computing the interaction contrast (Z[FS-NS]-[FNS-NNS] = ZFS-NS – ZFNS-NNS). Subsequently, we conducted two-sample t-tests on each pair of network-level functional connectivity to determine the treatment effect*.*

**Development and evaluation of CAFE^5^**

The CAFE (a synergistic brain connectivity- and activity-based signature for subjective fear) has been developed to accurately track subjective fear in dynamic naturalistic based on fMRI activation and connectivity patterns, and has been extensively evaluated in our previous work **^5^**. In this study, 76 participants underwent 38 short fear-inducing video clips over 4 runs, the participants were instructed to attentively watch the videos and rate their level of fear experience following each video. The videos lasted 30 - 50 s followed by a 1 - 1.5 s jittered fixation cross separating the stimuli from the rating period. During the subsequent 5 s period participants reported the level of fear they experienced for each stimulus using a 9-point Likert scale with 1 indicating no fear and 9 indicating very strong fear followed by a jittered 10 - 12 s inter-trial-interval (fixation-cross)*.*

The dataset was split into training (n = 51; i.e., 2/3 of the total participants) and test (n = 25) datasets. Next, the researcher developed multivariate fear signatures through whole brain activation-based (incorporating activation for each voxel; n = 228,022 features), functional connectivity-based (including connectivity between each pair of parcels; n = 106,953 features), and synergistic (integrating mean activation for each parcel and functional connectivity between each pair of parcels; n = 107,416 features) approaches using the training data only (n = 341 samples). Following their previous studies^10, 11^, they applied the linear SVR algorithm with the cost parameter C = 1 and epsilon = 0.01 to develop the fear signatures. This algorithm was implemented in the CanlabCore toolbox (https://github.com/canlab/CanlabCore), which utilizes the Spider toolbox (http://people.kyb.tuebingen.mpg.de/spider). The atlas-based parcellation used to develop connectivity-based and synergistic signatures were the totally same as our work, which included 463 regions across the whole brain. To evaluate model performance, they assessed the prediction-outcome correlation and coefficient of determination (R^2^) within the training data using cross-validated analysis (10 repeats of 10-fold cross validation), as recommended by refs.^12, 13^ To avoid the potential bias in train-test split, they re-ran this procedure 100 times.

In addition to cross-validating performance in the training data, the performance of the synergistic signature (i.e., the CAFE) for predicting fear were further tested in independent test data including (Thirty-six participants (17 females; age = 21.03 ± 2.22) watched a segment from the horror movie “The Conjuring 2” ( duration :11 min 18 s) with no response to facilitate a naturalistic and ecologically valid emotional experience; 31 participants (15 females; age = 23.29 ± 4.21) underwent a 1-hour fMRI session in which the participants were presented with 3600 images consisting of 30 animal categories and 10 object categories (90 different images per category). The stimuli were grouped in blocks of 2, 3, 4 or 6 images of the same category with each stimulus presented for 1 s (no inter-block or inter-stimulus interval). Subjective fear ratings (0 = ‘no fear’ to 5 = ‘very high fear’) for each category were established before the fMRI procedure without presenting any fearful stimuli). Significance testing was conducted using one-tailed permutation test: P = (1+number of r/R^2^ >= empirical r/R^2^)/(1+number of permutations).

The CAFE accurately predicted the stable subjective fear experience elicited by movie clips, both in the training dataset (cross-validated prediction-outcome correlation r = 0.64, R^2^ = 0.40, both permutation P < 10^-4^) and the test dataset (r = 0.67, R^2^ = 0.43, both permutation P < 10^-4^ ). The synergistic fear model, CAFE, surpassed the conventional whole-brain voxel-wise activation-based signature (training data: r = 0.60, R^2^ = 0.33; test data: r = 0.62, R^2^ = 0.36) to a varying degree, with the differences of R2 achieving (marginal) significance (training data: permutation P = 0.006; test data: permutation P = 0.085). The CAFE model exhibited a significant performance to accurately differentiate fear from other emotions (accuracies ≥ 84.17%, P < 10^-14^)*.*

Supplementary Results

The selected video clips were sufficient to induce corresponding emotions on both behavioral and neural level

In the pilot study, compared to neutral clips, the selected fear video clips (FS and FNS) yielded considerably higher fear ratings, and the main effects of emotion and social were significant (*F*_(1,18)_ = 290.6, *p <* 0.0001；*F*_(1,18)_ = 25.92, *p*＝0.001, **Fig. S3a**), the interaction between emotion and social was also significant(*F*_(3,18)_ = 42.81, *p =* 0.0001, **Fig. S3a**), and no sex differences were found in both sex-related main effects and interaction effects (*all ps >* 0.2333, **Fig. S3a**). No main effects of social were found (all *p*s > 0.05, **Fig. S3c** and **Fig. S3e**) and the main effects of emotion were both significant in arousal and valence ratings(*F*_(3,18)_ = 370.4, *p <* 0.0001, **Fig. S3c,** *F*_(1,18)_ = 72.71, *p <*0.0001, **Fig. S3e**), females tended to rate lower than males on arousal ratings (*F*_(1,18)_ = 16.01, *p =* 0.0031, **Fig. S3c**), in addition, the interaction between emotion and social was also significant(*F*_(3,18)_ = 10.71, *p =* 0.0096, **Fig. S3a**). Social video clips (FS and NS) yeileded much higher social ratings compared to non-social ones, the main effect of social was significant (*F*_(1,18)_ = 611.2, *p* < 0.0001, **Fig. S3b**) and no other significant main effects and interaction effects. Lastly, all selected video clips with pretty high consistency rating scores (see **Fig. S3d**). Detailed rating information about the slected clips in each condition were listed in **Table. S1**.


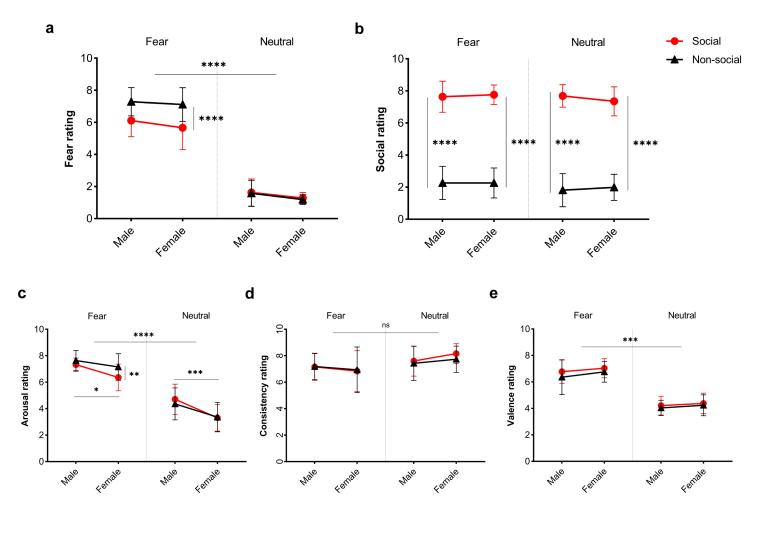


**Fig. S3 The selected stimuli for fMRI experiment were sufficient to induce corresponding emotions.** **a** The participants tended to rate subjective fear lower on fear social clips compared to their non-social peers in both gender, but this difference didn’t survive on **e** valence rating and **c** arousal rating (only in female, so we recruited only males in the latter fMRI study to remove the possible confounding roles and gender differences). **b** The selected social stimuli all have much higher rating scores on social than non-social controls. In addition, all selected stimuli have **d** pretty high consistency rating scores.

Table. S1 Detailed information about ratings of the selected stimuli

| Condition | Fear (M$\pm$SD） | Arousal (M$\pm$SD） | Valence (M$\pm$SD） | Social (M$\pm$SD） | Consistency (M$\pm$SD） |
| --- | --- | --- | --- | --- | --- |
| FS | 5.89$\pm$1.19 | 6.84$\pm$0.92 | 6.81$\pm$0.79 | 7.70$\pm$0.79 | 7.00$\pm$1.29 |
| FNS | 7.20$\pm$0.95 | 7.39$\pm$0.90 | 6.56$\pm$1.07 | 2.26$\pm$0.96 | 7.06$\pm$1.37 |
| NS | 1.45$\pm$0.66 | 4.01$\pm$1.27 | 4.29$\pm$0.73 | 7.52$\pm$0.81 | 7.88$\pm$0.98 |
| NNS | 1.38$\pm$0.63 | 3.86$\pm$1.24 | 4.14$\pm$0.68 | 1.90$\pm$0.91 | 7.58$\pm$1.13 |

Values presented here as mean$\pm$standard deviation (M$\pm$SD). FS fear social, FNS fear non-social, NS neutral social, NNS neutral non-social.

In the fMRI study, the selected fear video clips (FS and FNS and their related contrasts, see **Table. S3**) elicited greater activation in fear processing regions (including the amygdala, insula and the cingulate cortex, which were marked in dark blue) compared to neutral clips. The neural activity induced for the social and non-social fear clips encompassed a set of distributed common regions as well as some distinct regions (see Fig. S4). Together these results confirm the selection of the video clips. Brief descriptions of each video clip used in the fMRI study are also provided in Table. S2. Details regarding the brain regions activated were presented in **Table. S3**.


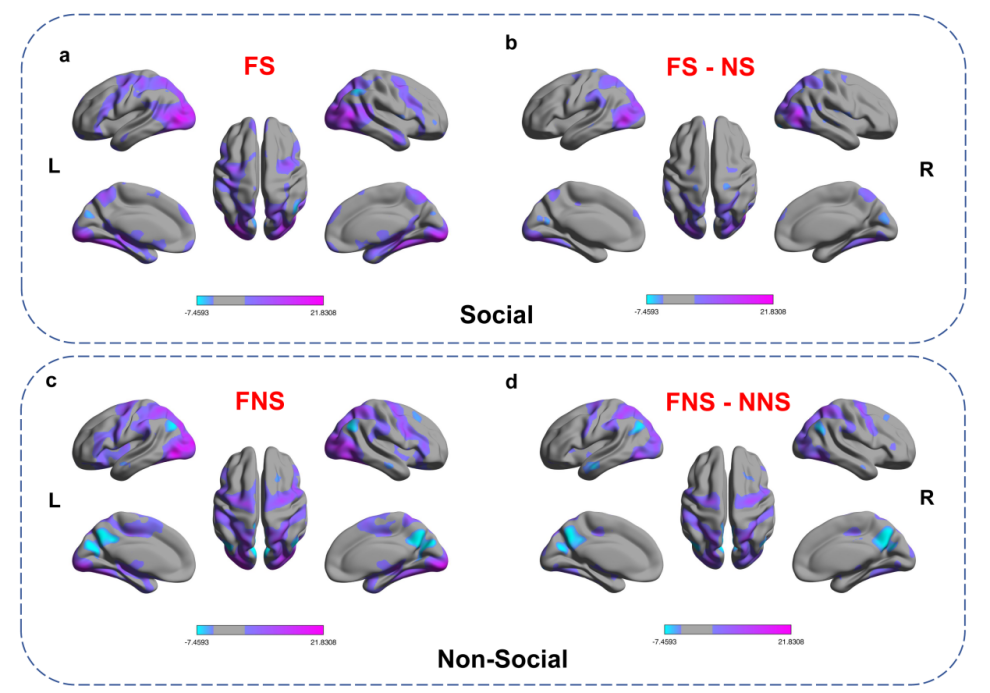


**Fig. S4 Shared and distinct activated neural patterns induced by the selected stimuli for fMRI experiment under social and non-social fear contexts.** All the results presented here came from only PLC group to see the overall activation induced by video clips and at peak-level unthreshold p < 0.001, FS fear social, FNS fear non-social, NS neutral social, NNS neutral non-social.

Table. S2 Detailed information about the selected stimuli

| Stimuli type | Number | Brief description |
| --- | --- | --- |
| FS | FS01 | Demonstrators clash with police in standoff, scene of flames |
|  | FS02 | Smoke billows from clashes between police and demonstrators |
|  | FS03 | Heavy clashes as many armed men ganged up on one man |
|  | FS04 | Two social gangs were fighting each other with weapons, and the scene was intense |
|  | FS05 | Very strong earthquake scene, people running for their lives |
|  | FS06 | Rescue after the earthquake, rescuers are passing through fast and dangerous rivers |
|  | FS07 | Firefighters work to extinguish a house fire as it rages on |
|  | FS08 | Firefighters work to extinguish a factory fire as it rages on |
| FNS | FNS01 | A man performing a dangerous maneuver at high altitude |
|  | FNS02 | A man running fast at high altitude from a first viewpoint |
|  | FNS03 | A python is strangling its prey and opening its bloody mouth to try and swallow it |
|  | FNS04 | A giant python is spitting on the riverbank and crawling closer |
|  | FNS05 | A giant spider crawls and feeds fast |
|  | FNS06 | A close-up of a giant spider and its feeding scene |
|  | FNS07 | First view of a fast ride in the very rugged outdoors |
|  | FNS08 | First view of a fast ride in the very rugged outdoors with body flips |
| NS | NS01 | Two students go up and explain while the teacher and classmates listen from below |
|  | NS02 | The teacher is giving a lecture and the students are listening carefully |
|  | NS03 | Two schoolgirls met and sat down to have a short chat |
|  | NS04 | A man and a woman were sitting on benches near the river and chatting face to face |
|  | NS05 | A chemist explaining science-related work to the public |
|  | NS06 | A physicist explaining science-related work to the public |
|  | NS07 | Hanging out at the mall in the first place |
|  | NS08 | Hanging out at the supermarket in the first place |
| NNS | NNS01 | A herd of cows is grazing and eating milk on the grassland |
|  | NNS02 | A herd of cows is grazing and resting in the pasture |
|  | NNS03 | A flock of seagulls circled along the highway by the sea |
|  | NNS04 | A flock of seagulls circled along the sandy beach by the sea |
|  | NNS05 | Calm lake and relaxed animals |
|  | NNS06 | Calm lakes and quiet woods |
|  | NNS07 | Greenery swaying in the breeze and beautiful sunsets |
|  | NNS08 | Pieces of wheat swaying in the breeze |

Table. S3 Activated brain regions under fear-related conditions in PLC group

| Condition\  contrast | brain region | MNI(x,y,z) | cluster level | | | | peak level | | | |
| --- | --- | --- | --- | --- | --- | --- | --- | --- | --- | --- |
|  |  |  | *p*_FWE-corr_ | *P*_uncorr_ | k | *p*_FWE-corr_ | | *P*_uncorr_ | *t* | *Z* |
| FS | RH-lingual gyrus extending to **amygdala** | 18, -83, -13 | 0.000 | 0.000 | 424820 | 0.000 | | 0.000 | 21.83 | Inf |
|  | **RH-insula** | 58, -1, -17 | 0.000 | 0.000 | 467 | 0.000 | | 0.000 | 10.71 | 6.99 |
|  | RH-dlPFC | 42, 2, 50 | 0.000 | 0.000 | 1987 | 0.000 | | 0.000 | 9.97 | 6.72 |
|  | RH-OFC | 38, 24, -31 | 0.000 | 0.000 | 487 | 0.000 | | 0.000 | 8.59 | 6.18 |
|  | LH-vlPFC  extending to **insula** | -57, 28, 22 | 0.000 | 0.000 | 717 | 0.000 | | 0.000 | 8.38 | 6.1 |
|  | **RH-ACC** | 6, 52, 34 | 0.000 | 0.000 | 631 | 0.001 | | 0.000 | 7.33 | 5.61 |
|  | LH-OFC | -33, 34, -23 | 0.000 | 0.000 | 393 | 0.000 | | 0.000 | 6.91 | 5.4 |
| FNS | LH-lingual gyrus extending to **amygdala** | -9, -85, -11 | 0.000 | 0.000 | 406620 | 0.000 | | 0.000 | 22.21 | Inf |
|  | RH-dlPFC | 50, 10, 32 | 0.000 | 0.000 | 5548 | 0.000 | | 0.000 | 12.65 | 7.58 |
|  | LH-dlPFC | -59, 10, 34 | 0.000 | 0.000 | 701 | 0.000 | | 0.000 | 9.29 | 6.47 |
|  | LH-vlPFC  extending to **insula** | -55, 34, 22 | 0.000 | 0.000 | 380 | 0.000 | | 0.000 | 8.19 | 6.01 |
|  | RH-STG | 62, -39, 18 | 0.000 | 0.005 | 61 | 0.000 | | 0.000 | 7.58 | 5.73 |
|  | LH-PHG | -15, -25, 33 | 0.000 | 0.008 | 52 | 0.005 | | 0.000 | 6.6 | 5.23 |
| FS-NS | LH-MOG | -45, -73, 4 | 0.000 | 0.000 | 5564 | 0.000 | | 0.000 | 16.1 | Inf |
|  | RH-MOG | 48, -67, -1 | 0.000 | 0.000 | 4683 | 0.000 | | 0.000 | 15.92 | Inf |
|  | RH-SPG | 30, -57, 60 | 0.000 | 0.000 | 931 | 0.000 | | 0.000 | 9.27 | 6.46 |
|  | LH-SPG | -25, -61, 58 | 0.000 | 0.000 | 765 | 0.000 | | 0.000 | 8.6 | 6.19 |
|  | RH- cerebellum | 16, -51, -51 | 0.000 | 0.000 | 213 | 0.000 | | 0.000 | 8.31 | 6.06 |
|  | LH- cerebellum | 16, -81, -51 | 0.000 | 0.000 | 157 | 0.000 | | 0.000 | 8.06 | 5.95 |
|  | LH-precuneus | -9, -59, 50 | 0.000 | 0.001 | 83 | 0.003 | | 0.000 | 6.86 | 5.37 |
|  | LH-SMG | -63, -29, 38 | 0.000 | 0.000 | 136 | 0.003 | | 0.000 | 6.85 | 5.36 |
| FNS-NNS | RH-SPL | 28, -63, 66 | 0.000 | 0.000 | 171510 | 0.000 | | 0.000 | 15.86 | Inf |
|  | RH-FEF\dlPFC | 28, -11, 58 | 0.000 | 0.000 | 1463 | 0.000 | | 0.000 | 11.59 | 7.27 |
|  | LH-dlPFC | -23, -9, 66 | 0.000 | 0.000 | 834 | 0.000 | | 0.000 | 10.23 | 6.82 |
|  | LH-Hippocampus | -21, -31, 8 | 0.001 | 0.011 | 47 | 0.000 | | 0.000 | 7.85 | 5.86 |
|  | **LH-amygdala** | -9, -11, -13 | 0.001 | 0.02 | 38 | 0.004 | | 0.000 | 6.7 | 5.29 |
|  | RH-red nucleus | 4, -25, -5 | 0.000 | 0.001 | 102 | 0.005 | | 0.000 | 6060 | 5.25 |

*p*_FWE-corr_ represented thresholds results p < 0.05(FWE Multiple Comparison Correction), and for display purpose only cluster size k > 30 was listed here. FWE, family wise error.

OT enhanced FPN-DAN and DMN-DAN coupling during fear as modelled by an interaction contrast

Examining the effects of OT on the large-scale network level using separate two-sample t-tests on the matrices of z-values for the fear interaction contrast ([FS-NS]-[FNS-NNS]) revealed that compared to PLC, OT significantly enhanced the functional connectivity between FPN and DAN (*t*_(1,65)_$=$2.96, *p*$=$0.0043, **Fig. S5**) as well as FPN and DMN’S control on SMN (FPN-SMN: *t*_(1,65)_$=$2.34, *p*$=$0.0224; DMN-SMN: *t*_(1,65)_$=$2.00, *p*$=$0.0492, **Fig. S5**), and a marginally significant functional coupling between DMN and DAN (*t*_(1,65)_$=$1.66, *p*$=$*0.*1024, **Fig. S5**) in the fear interaction context. In line with the social context specific analyses presented in the main manuscript these results further underscore that OT’s network level effects depend on the social versus non-social context.


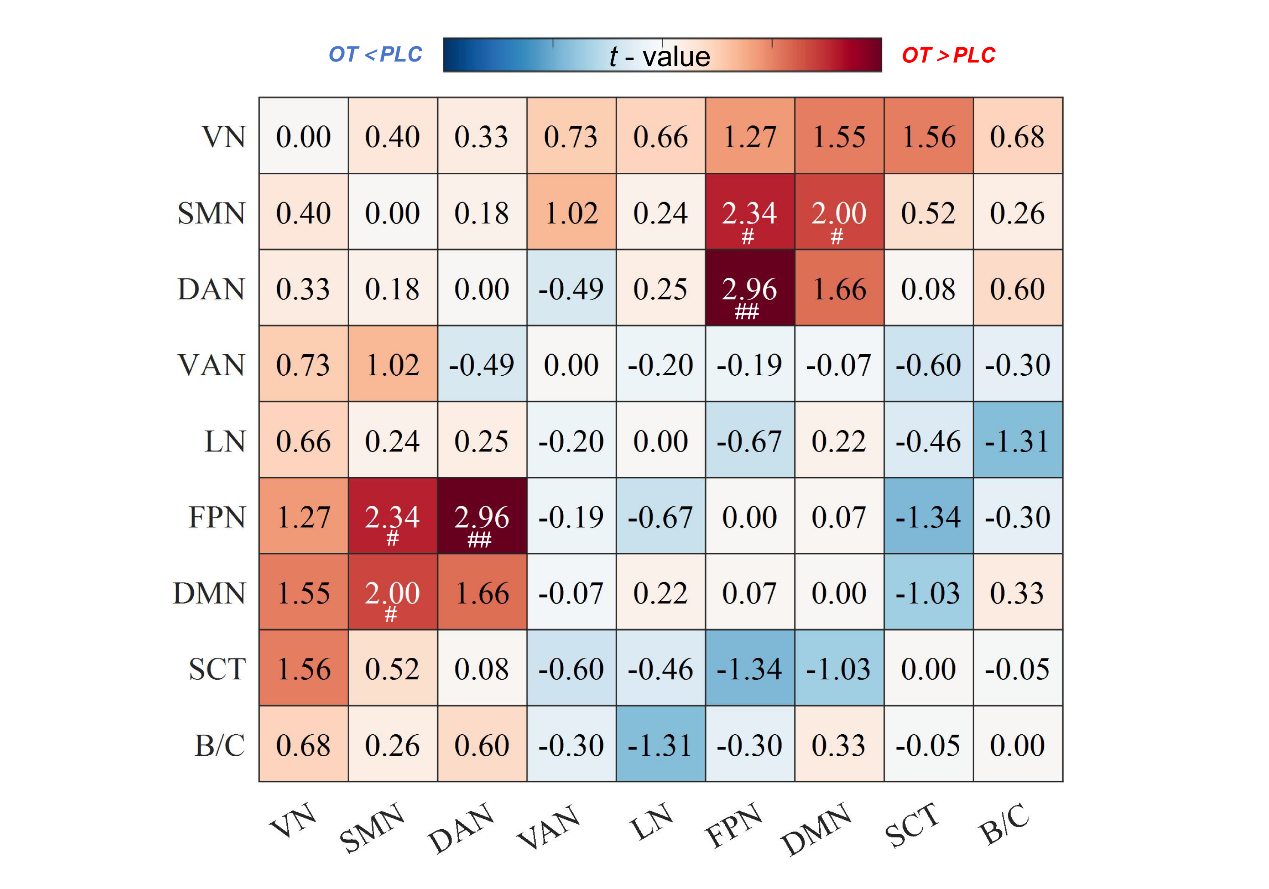


**Fig. S5 OT consistently enhanced functional connectivity between FPN and DAN on the level of large-scale networks**. OT significantly strengthened the functional connectivity between FPN and DAN and nearly significant functional connectivity between DMN and DAN besides the functional coupling between FPN and SMN under fear interaction contexts. OT oxytocin, PLC placebo, FS fear social, FNS fear non-social, NS neutral social, NNS neutral non-social, VN visual network, SMN somatomotor network, DAN dorsal attention network, VAN ventral attention network, LN limbic network, FPN frontoparietal network, DMN default mode network, SCT subcortical network, B/C brainstem and cerebellum. ##*p*_un-corrected_$<$0.01, # *p*_un-corrected_$<$0.05.

References

1. Avants BB, Tustison N, Song GJTIJ. Advanced Normalization Tools: V1.0. 2009.

2. Zhang Y, Brady M, Smith S. Segmentation of brain MR images through a hidden Markov random field model and the expectation-maximization algorithm. *IEEE Transactions on Medical Imaging* 2001; **20**(1)**:** 45-57.

3. Cox RW. AFNI: Software for Analysis and Visualization of Functional Magnetic Resonance Neuroimages. *Computers and Biomedical Research* 1996; **29**(3)**:** 162-173.

4. Greve DN, Fischl B. Accurate and robust brain image alignment using boundary-based registration. *NeuroImage* 2009; **48**(1)**:** 63-72.

5. Feng Z, Ran Z, Shuxia Y, Debo D, Pan F, Georg K *et al.* Capturing dynamic fear experiences in naturalistic contexts: An ecologically valid fMRI signature integrating brain activation and connectivity. *bioRxiv* 2024**:** 2023.2008.2018.553808.

6. Schaefer A, Kong R, Gordon EM, Laumann TO, Zuo X-N, Holmes AJ *et al.* Local-Global Parcellation of the Human Cerebral Cortex from Intrinsic Functional Connectivity MRI. *Cerebral Cortex* 2018; **28**(9)**:** 3095-3114.

7. Tian Y, Margulies DS, Breakspear M, Zalesky A. Topographic organization of the human subcortex unveiled with functional connectivity gradients. *Nat Neurosci* 2020; **23**(11)**:** 1421-1432.

8. Pauli WM, Nili AN, Tyszka JM. A high-resolution probabilistic in vivo atlas of human subcortical brain nuclei. *Scientific Data* 2018; **5**(1)**:** 180063.

9. Lee J-J, Kim HJ, Čeko M, Park B-y, Lee SA, Park H *et al.* A neuroimaging biomarker for sustained experimental and clinical pain. *Nature Medicine* 2021; **27**(1)**:** 174-182.

10. Zhou F, Zhao W, Qi Z, Geng Y, Yao S, Kendrick KM *et al.* A distributed fMRI-based signature for the subjective experience of fear. *Nature Communications* 2021; **12**(1)**:** 6643.

11. Gan X, Zhou F, Xu T, Liu X, Zhang R, Zheng Z *et al.* A neurofunctional signature of subjective disgust generalizes to oral distaste and socio-moral contexts. *Nature Human Behaviour* 2024; **8**(7)**:** 1383-1402.

12. Poldrack RA, Huckins G, Varoquaux G. Establishment of Best Practices for Evidence for Prediction: A Review. *Jama Psychiat* 2020; **77**(5)**:** 534-540.

13. Scheinost D, Noble S, Horien C, Greene AS, Lake EMR, Salehi M *et al.* Ten simple rules for predictive modeling of individual differences in neuroimaging. *NeuroImage* 2019; **193:** 35-45.
